# Author Correction: High-throughput microCT scanning of small specimens: preparation, packing, parameters and post-processing

**DOI:** 10.1038/s41598-020-77000-6

**Published:** 2020-11-11

**Authors:** Christy A. Hipsley, Rocio Aguilar, Jay R. Black, Scott A. Hocknull

**Affiliations:** 1 School of BioSciences, University of Melbourne, BioSciences 4, Building 147, Parkville, VIC 3010 Australia; 2 Museums Victoria, GPO Box 666, Melbourne, VIC 3001 Australia; 3 School of Biological Sciences, Monash University, Clayton, VIC Australia; 4 School of Earth Sciences, University of Melbourne, Melbourne, VIC Australia; 5 Queensland Museum, Geosciences, 122 Gerler Rd., Hendra, QLD 4011 Australia

Correction to: *Scientific Reports* 10.1038/s41598-020-70970-7, published online 17 August 2020

In this Article, the legend of Figure 4 is incorrect:

“(**c**) mandible of the rainforest rodent *Pogonomys* from the Riversleigh World Heritage site with molars colour coded and jaw bone rendered semi-transparent,”

should read:

“(**c**) mandible of the rainforest rodent *Pogonomys* from the Mount Etna Caves National Park with molars colour coded and jaw bone rendered semi-transparent,”

